# Law, Philosophy and the Susceptible Skins of Living Beings

**DOI:** 10.1093/ojls/gqaf022

**Published:** 2025-06-20

**Authors:** David Enoch

**Keywords:** legal philosophy, legal theory, methodology, legal epistemology

## Abstract

Catherine the Great (apparently) wrote to the French philosopher Diderot something along the lines of: ‘You philosophers are fortunate. You write on paper, and paper is patient. Unfortunate emperor that I am, I write on the susceptible skins of living beings.’ Catherine expressed, I think, an important insight, that is true of the law as well: the law writes on the susceptible skins of living beings. This does not mean, of course, that we should not philosophise about the law, or that we should not take advantage of the benefits of having patient paper to write on. But as we do so, we should philosophise about the law all the time fully realising that the law itself does not write on patient paper, but on the susceptible skins of living beings. This has important implications to how we should—and how we should not—do philosophy of law. This article elaborates on these implications—both in general and using more specific examples.

You philosophers are fortunate. You write on paper, and paper is patient. Unfortunate Empress that I am, I write on the susceptible skins of living beings.

(Catherine the Great, I think^1^)

## 1. Introduction: The Import Temptation

The law often discusses causation. It matters greatly—for the theory of criminal law, for the theory of tort law, indeed for courts deciding cases across legal areas—how causation is best understood. Metaphysicians also discuss causation^2^—its nature, how, if at all, we can know about it, and so on. Some of these metaphysical discussions are quite impressive. When it comes to the nature of causation or the necessary and sufficient conditions for one event to cause another, metaphysicians are typically much better than lawyers, and indeed than legal theorists. And so there is the temptation to import the metaphysicians’ lessons into legal theory, and indeed to the law: if causation is best thought of in probabilistic terms, say, or counterfactually, if causal contribution comes in degrees^3^ or if we know something in general about causal overdetermination, we can just import this knowledge of causation into causation in the law, and thereby make progress not only on the understanding of legal causation, but also on deciding legal cases in a more defensible way.

The law often invokes knowledge claims. This is perhaps most common in evidence law—or at least, evidence law *theory*—but it is quite common elsewhere as well, as when the law is interested in what different agents knew or should have known. Epistemologists also discuss knowledge, and other, related concepts like those of evidence, justification, warrant. Such epistemological discussions can be quite impressive. Indeed, when it comes to the nature of knowledge, or the necessary and sufficient conditions for knowledge, epistemologists are typically much better than lawyers or even legal theorists. So there is the temptation to import the epistemologists’ lessons into legal theory, and indeed into the law: if knowledge requires the elimination of relevant alternatives, if statistical evidence never suffices for justified belief and for knowledge or if knowledge requires that luck was not involved in the formation of the relevant true belief, we can just import this understanding of knowledge and evidence into knowledge and evidence in the law, and thereby make progress not only on the theoretical understanding of what is now often called ‘legal epistemology’,^4^ but also on deciding legal cases in a more defensible way.

And you see how it goes—whatever you lawyers and legal theorists want, pretty much, we philosophers have something to offer, something for you to import. If, for instance, you are interested, in a legal or legal-theoretical context, in what amounts to an action, in the distinction between actions and omissions or in the nature of attempts, then philosophy of action has something for you to import. Philosophers of action, after all, are quite impressive in discussing the nature of action, more so than legal theorists. Or perhaps you are discussing the liability of corporations or legal interpretation, and so are interested in whether or not corporations and the legislature have intentions and other mental states. Surely, the place to go to is the philosophy of mind, where you can find the most impressive discussions of the nature of such mental states, and indeed of the necessary and sufficient conditions for having, say, an intention. You can then import the lessons of these discussions into your discussions of corporate intentions and legislative intent.^5^ Or perhaps you are interested—perhaps still in the context of legal or constitutional interpretation—in the nature of meaning and interpretation. Why not turn, then, to the philosophy of language, where such matters receive the best, most rigorous treatment, and then import whatever you find there into legal theory, and indeed the law?

This, then, is what I call *the import temptation*, and as my name for it suggests, I will argue that it is important that we resist it. My point here is not the rather obvious but limited one, namely, that rarely, if ever, do philosophical discussions (in metaphysics, epistemology, the philosophy of language or …) conclude in a consensual claim that can then be taken as a given for less philosophical contexts. Rather, I argue that the thought that we can unproblematically import lessons from philosophy to law (or legal theory) in this way ignores a crucial difference in the nature of the two projects, a difference that Catherine the Great seems to have been clear on.

Section 2 is the negative heart of the article, containing the main argument against the import temptation. In the following section, I address an important objection to my argument—the thought that because both law and philosophy use natural language terms, importing lessons from philosophy to law has to be legitimate. Section 4 offers positive alternatives: if the import temptation has to be resisted, how *can* philosophy be relevant and helpful for the law and for legal theory? Section 5 is more abstract. In it, I examine in a preliminary way possible generalisations of the lessons of previous sections. Such generalisations include questions about both how *other things* (like empirical science) matter to the law and how *philosophy* can matter *to other things* (like morality). I conclude with a short, self-referential reflection.

Before continuing, though, I want to emphasise a point that will re-emerge from time to time below. By rejecting the import temptation, I will be in effect arguing that there is this *one way* in which you may have thought that philosophy mattered to the law, but that in fact it does not. I will not, however, claim that the law—or legal theorists, or faculties of law!—should not care about philosophy in general, or that philosophy does not matter to the law. If successful, the upshot of this article will be not the debunking of legal philosophy, but a distinction between good and bad ways of doing legal philosophy. Indeed, I am a philosophical imperialist—I believe that philosophy, done well, is relevant to *everything*, and that there is often no way of doing at least normative legal theory without doing philosophy. The way we should do it, though, is not the way that succumbs to the import temptation. Or so I shall argue.

## 2. The Mistake; or, how Catherine was Right

Suppose you are a judge—say, in an appellate court^6^—about to decide a case. Your decision will, as many such decisions do, have real effects on the lives of real people. Suppose that the decision will turn on how, in this context, knowledge and evidence are to be understood—perhaps because whether the defendant is liable depends on whether she knew or should have known something, or perhaps because your decision may depend on whether the evidence in front of you suffices for some relevant finding. Making legal decisions may be complicated. Many considerations may be relevant here. You may want to consider such questions as whether anyone’s legal, and perhaps also moral, rights were violated; what the effects of different findings will be on the plaintiff and on the defendant; what, if anything, you owe the legislature here, or any other relevant institutional aspects; what the wider consequences will be—say, in generating incentives for behaviours of many who are not parties to the current proceedings; and so on. The relevant considerations may vary from case to case, and each one of them may be controversial (perhaps in some proceedings, in some instances, nothing about wider incentives and deterrence is a legitimate reason for a decision). None of these complications matter for our purposes here. All that matters is that many considerations may be relevant for deciding such cases.

But now suppose that your clerk comes along with breaking news from the epistemology seminar room. You cannot know that p—epistemologists have now come to realize —unless the fact that p played an appropriate causal role in bringing about your belief that p!^7^ Or perhaps the degree of evidential support needed for knowledge that p varies according to the practical significance of p (in the circumstances).^8^ Remember—you are the judge, trying to reach a decision in a case that will affect the lives of real people, and many practical considerations are relevant. Can you imagine a situation in which, say, you are about to find for the plaintiff, but the breaking news from the epistemology seminar room, all by itself,^9^ (rationally) changes your mind and you find for the defendant? Can you imagine any situation in which the news from the epistemology seminar room—about the nature of knowledge, or evidence—somehow generates reasons for deciding in one way that outweigh any of the practical considerations mentioned in the previous paragraph?

To me, this sounds both descriptively and normatively ridiculous. Descriptively, I find it very hard to believe that any judge has ever been affected in this kind of way by the breaking news from the epistemology seminar room. Now, this is an empirical question, so my point here is speculative, and I guess we have to await the empirical findings on this one (still, once qualified to make room for the occasional judge who falls prey to the import temptation, this is a speculative point I remain very confident in). Normatively, though, I do not think we need to wait for anything. No good judge is willing to let philosophical niceties of the kind just sketched,^10^ all by themselves, affect a decision that affects the lives of real people. Whatever other, practical considerations a good judge may be moved by, the breaking news from the epistemology seminar room is not one of them.^11^

But—you may be tempted to think—how can a good judge *avoid* being affected by the breaking news from the epistemology seminar room, seeing that the law refers to *knowledge* and *evidence*, and we just got news precisely about knowledge and evidence? Well, good judges—and rather mediocre ones too—have no problem disambiguating.^12^ If need be, they can say, or even just think, something like: well, sure, those epistemologists are really very smart, and they may have found out something about the nature of knowledge and evidence. But *for legal purposes*, knowledge should be understood differently. Indeed, perhaps even more finely, *for the purposes of tort liability*, evidence should be understood to require this or that condition; or perhaps even—*for the purpose of this specific case*, this is how we should understand knowledge. (I will not say anything on what the appropriate level of generality is here; I suspect it varies widely with context.) A striking feature of this disambiguation move is that it comes so naturally to judges, as indeed it should. It is not as if this is something a theorist with too much free time came up with in order to save her theory—it is a standard judicial move that everyone involved with the law is familiar with instances of.

If we want to put things more precisely—but this will no longer be a standard judicial move—we could put it this way: upon receiving the breaking news from the epistemology seminar room, the judge distinguishes between two senses of ‘knowledge’—the one the philosophers are interested in, which we can call Knowledge_E_, and the one relevant to law (or to some specific part thereof), which we can call Knowledge_L_. The judge does not dispute, then, the philosophical achievement—the news from the epistemology seminar room may very well tell a new exciting story about Knowledge_E_. But it does not tell a story about Knowledge_L_, at least not directly. Knowledge_E_ and Knowledge_L_ may come apart. Knowledge_E_ is governed by the kind of considerations philosophers take seriously in the epistemology seminar room. Knowledge_L_, on the other hand, is governed by the many and varied practical considerations that are relevant to legal decisions. It is possible, of course, that Knowledge_E_ and Knowledge_L_ overlap, or, indeed, that they fully coincide. But it is not necessary, and if you want to move from claims about Knowledge_E_ to ones about Knowledge_L_, you have to show that such a move is legitimate (there is more below on how this can sometimes be shown). You do not get it, as it were, for free, just because we use the word ‘knowledge’ for both.

With or without such a disambiguation move, then, courts should be indifferent to the breaking news from the epistemology seminar room.^13^ And this is an indication of a wider point here: had it been the case that the law was supposed to be sensitive to the kind of knowledge discussed in epistemology, or to the kind of considerations that govern it—had the law, as we might put it, cared about Knowledge_E_—it *would* have been sensitive to the breaking news from the epistemology seminar room. But it is not, and nor should it be. So the law, in a slogan, should not care about knowledge, thus understood. Knowledge_E_ does not matter to the law.^14^

Neither knowledge nor evidence is special in this way. The breaking news from the metaphysics seminar room is not more exciting, legally, than that from the epistemology seminar room, and with regard to causation, too, a good judge can distinguish between causation in general and causation as relevant to the law or to the case at hand, or between Causation_M_ and Causation_L_. And as before, the two may be related, but then again, they may not, and whether they are is not something we can decide by fiat, simply because we often use the same term (‘causation’) for both.^15^ The same goes for news from the philosophy-of-mind seminar room about intentions, and a possible distinction between Intention_P_ and Intention_L_, and for news from the philosophy-of-language seminar room about, say, meaning and interpretation, and a possible distinction between Meaning_P_ and Meaning_L_, and between Interpretation_P_ and Interpretation_L_.^16^ The same holds, as far as I can see, for pretty much any other piece of exciting breaking news from pretty much any other philosophy seminar room (with the possible exception of ethics and political philosophy; more on this below).^17^ And although the thought experiment I have been using has been about a judge, the lesson generalises—it is very hard to see why any other part of a legal system, or indeed *the legal system* itself, should care more about such breaking news.^18^

None of this means that philosophy should not matter to the law. I think that it should, and say more about this below. But if it does, the *way* in which it does is not one that entails that judges should simply decide cases based on the breaking news coming out of those seminar rooms.^19^

So far, this was, to put it roughly, about what *the law* should care about, for instance via thoughts about which considerations should move judges deliberating about deciding a case. But if what I have said so far is true—if the law should not in this way care about philosophy—this puts a very low upper bound on how helpful such philosophy can be in *legal theory* as well. This is because offering theories that make use of concepts, properties and categories that the law neither should nor does care about is highly unlikely to promote understanding of the law. In order to show this, it is helpful to use a more detailed example. Here comes a detour, then, on statistical evidence.^20^

A bus causes harm, and we have two cases. In the first case, there is an eyewitness testifying that the bus was blue. The eyewitness, we have independent reason to believe, is about 70% reliable on such matters in such circumstances. With a balance-of-probabilities burden of proof, the law has no problem grounding a finding in tort against the Blue Bus Company. In the second case, there are no eyewitnesses, and the only relevant evidence—uncontested, as it happens—is that the Blue Bus Company controls about 70% of the market at the relevant location, that is, about 70% percent of the buses passing through the relevant place, perhaps at the relevant time, belong to the Blue Bus Company. In this case, the law—across jurisdictions as far as I know—is *not* happy to issue a finding in torts, despite the balance-of-probabilities burden of proof. The theoretical problem here, of course, is to explain, and perhaps also justify, the distinction between the two cases.

One line of thought—perhaps more and more influential in the voluminous current literature in legal epistemology—suggests answers in epistemological terms.^21^ Perhaps, for instance, statistical evidence—as in the second case above—cannot support knowledge, and perhaps this is what explains the difference between the legal attitude to the two cases.^22^ Or perhaps we should design our legal procedures in a way that makes achieving knowledge of the relevant facts most likely, and the fact that statistical evidence cannot generate knowledge is therefore the reason we should be suspicious of it.^23^ Or something along these lines.

But now we know better. First, no breaking news from the epistemology seminar room (say, about how in some cases statistical evidence can after all suffice for knowledge!) should change any judge’s mind about how to decide any of the Blue Bus cases. This was the point emphasised above. But also, second, this means that there is very limited role for knowledge—or anyway, for Knowledge_E_—in *the theory of* statistical evidence. In particular, if the law should not care in this way about Knowledge_E_, then no theory of the difference between the two Blue Bus cases that relies heavily on the concept of Knowledge_E_ can *justify* the law’s differential treatment of the two cases. This does not mean such a theory has no value at all—it can still *explain* the differential treatment. But this would be a debunking explanation, not a vindicating one. Consider, for analogy: suppose we are trying to explain why a certain professor marked one group of essays higher than another. Suppose the explanation is in terms of how confident the writing tone is. And suppose further that we rightly believe that a confident tone should not matter to essay marks. In that case, the story in terms of the confident tone may yet be a good *explanation* of the professor’s differential marks, but it cannot be a *justification* thereof. It can only be a debunking explanation—a story explaining the differential marking that presents it in a poor, normatively unflattering, unjustifying light. The same holds for a knowledge-based explanation of the law’s differential treatment of statistical and (for instance) eyewitness testimony, if I am right that the law should not care about knowledge. But when we wonder about statistical evidence, what we want—most of us, anyway—is not just to explain the law’s treatment of statistical evidence, but also to vindicate it. This, a knowledge-based explanation cannot do.^24^

End of the statistical evidence detour. The lesson I want to draw from it is that if, as I have argued, some news from some philosophical seminar room should not matter to the law, it for the most part should not matter for legal theory either (except if in the relevant context we are in the business of offering a debunking explanation, or are at least open-minded about doing so).^25^ When it comes both to the law and to legal theory, the import temptation should be resisted.

One way of reading the quote from Catherine the Great that I started with is realising precisely this. (Of course, I have neither exegetical nor historical pretensions here.^26^) The law, like Catherine herself, writes on the susceptible skins of living beings. It affects the lives of real people. And so the set of considerations that govern it are practical ones.^27^ The set of considerations governing philosophical discussions are very different, because philosophy does not typically write on the susceptible skins of living beings, and the philosopher’s paper is, precisely as Catherine says, quite patient. So if, in order to achieve a better result in practical terms, the law must speak in a way that is not exactly philosophically precise, or it must show some indifference to the latest news from the philosophy seminar room or must speak about Causation_L_ and not Causation_M_, it is a very small price to pay. In fact, it is no price at all.^28^ The legal project and the philosophical ones are very different in nature, and so very different in the normative considerations that should guide them, precisely in the way (this understanding of) Catherine’s catchy metaphor captures.

Note, then, that the problem with legal philosophers who succumb to the import temptation is not that their work is too abstract. There is nothing wrong with doing absolutely abstract philosophy, about the law as about anything else. The problem is, first and very clearly, that if you do abstract philosophy on patient paper, and still somehow based on it hope to generate recommendations as to how the law should write on the susceptible skins of living beings, your reasoning is fallacious. And second, and more deeply, if you are philosophising about the law while ignoring the fact that it writes on the susceptible skins of living beings, you are missing one of the most important features of the phenomenon you are supposed to be philosophising *about*.^29^ The failure is not just the practical one—that no conclusions about, say, legal reform yet follow. The deeper flaw is *theoretical*—no understanding of the law, or of a specific legal doctrine, can arise from a story about the law that neglects the fact that the law, unlike philosophy, writes on the susceptible skins of living beings.

Before concluding this section, I want to make a concessive point about, well, references: throughout this section, and indeed throughout this article, I refer to examples of instances of the legal theorists seemingly succumbing to the import temptation. But such matters are rarely simple. Precisely because succumbing to the import temptation is fallacious, whenever an alternative reading of the relevant theorist is possible, one that does not have them commit this fallacy, this alternative reading will be the more charitable one. Thus, it will often be more charitable to read the relevant theorist as avoiding the fallacy (perhaps by endorsing, if only implicitly, the kind of bridging principle I proceed to discuss in section 4D below.^30^ For every specific example, determining what the best reading is would require a detailed engagement with the text (to see whether textual evidence counts sufficiently strongly against the more charitable reading). But such detailed engagement is beside the main point of this article, of course. So all references to theorists succumbing to the import temptation—throughout the article—should be taken as qualified. They are all examples of what at least *seems like* succumbing to the import temptation. My main point here, of course, is to warn against succumbing to the temptation, not in ‘catching’ anyone who has.^31^

## 3. But: Natural Language

You may think, though, that my argument against the import temptation has been resting on a false picture of how language works. When the law speaks, say, of intentions, it does not speak of the stipulated term Intention_L_. Nor does it speak, of course, about Intention_P_. Rather, it speaks of *intentions*, that is, of what we use the natural-language term ‘intention’ to pick out (I will use, as you might expect, subscriptless words to express their natural-language meaning or to refer to it; sometimes, in order to emphasise that it is the natural-language meaning I want to be using, I will use an NL-subscript, as in Intention_NL_). Similarly, when philosophers are out to understand intentions (or actions, causation, meaning, knowledge, evidence …) they too are after a better understanding of the thing we all talk about when we use these terms in our natural language. The point here can be seen as relying on some deep picture of philosophy as the more rigorous continuation of common-sense thinking, but it need not: it is simply the (related) observation that however jargonesque our philosophical discussions become, the questions or underlying concerns that start them off are not typically as jargonesque. After all, the philosopher studying, say, causation does not want to *change the subject* when they come up with their theory, they typically want their theory to be *of causation*, not of something only described by a made-up term stipulated to mean whatever it is that the philosopher wants it to mean. But if philosophical accounts of causation are accounts of causation-as-understood-in-natural-language, and if when the law speaks of causation it uses the term in its natural-language meaning, then Causation_M_ and Causation_L_ are after all synonymous, and we can do away with the subscripts. If so, the disambiguation move is blocked, and the import temptation should be happily succumbed to.

Even before more theoretical discussion, we know that something *must* be wrong with this line of thought, because had it worked, a good judge *would* have had to change their mind about a decision on a case because of the breaking news from the metaphysics seminar room, and *that* is such an implausible result that we know something must have gone wrong with the reasoning leading to it, even before we know what. But let us now say what *is* wrong with this reasoning. I will make three points: a tentative one about the law and natural language; a tentative one about philosophy and natural language; and a not-so-tentative one about the core and the periphery of the relevant concepts.

First, it is not at all clear that the law *does* speak natural language. It is not uncommon for the legal definition of a term—in general, or in some specific legal context—to depart from its natural language meanings. Some anecdotes from the first year of law school are instances of this phenomenon—as, for instance, when the court interprets the word ‘document’ to include also a car chassis (for the purpose of the criminal offence of falsifying a document).^32^ True, the law certainly works in a natural-language environment: despite the fact that for each specific contested term, it is quite possible that its use in law differs from its use in natural language, it remains true that most of what the law says it says either in natural language or in some closely related idiolect thereof. Indeed, perhaps there is a sense in which law’s starting point is that of natural language. But the kind of claim made use of above—namely, that Causation_L_ and causation are one—is just false. And the way in which it may be true to say that law’s starting point is that of natural language does not even lend support to thinking that there is a presumption in favour of understanding legal terms in their natural language meaning.^33^

Next, philosophy: here, I think that the relation to natural language is closer. At least when done well, epistemology is not about developing a new concept, which we can call ‘knowledge’.^34^ It is about studying (among many other things) *knowledge*, the thing we have been talking about all along. In this way, the starting point of philosophy is natural language. But this does not mean that this is where philosophy ends up as well. In this respect, philosophy is like science. When biology studies, say, fish, it studies *fish*, the phenomenon we have been talking about using the natural-language term ‘fish’. But biology may end up suggesting rather revisionary claims—for instance, that a whale is not a fish. And based on the strength of the biological theories, we accept this surprising claim, and sometimes even revise our natural language use of the term accordingly. In a similar way, while philosophy starts from our natural language concepts, it may end up with some revisionary claims—perhaps, for instance, the best, most unified, most explanatory potent philosophical theory of causation will have to somewhat depart from common natural-language uses of relevant terms. That this can happen—that it often does—is one central way in which philosophy differs from lexicography.^35^

But third, and most importantly, even if in many cases a term has the same meaning across natural language, the law and philosophy, this will not apply precisely to the cases we are interested in, the cases about which we tend to theorise. The very fact that we need to theorise about some concept or about its application is evidence that it is not an unproblematically applied concept in an unproblematic context. If we had a clear case of someone neither knowing nor being required to know something, we would not have had to do theory—either legal theory or epistemology—in order to say that the ‘knew or should have known’ condition is not met here. The very fact that we need theory here shows that it is not a core case, but rather a peripheral one, a case in which it may be seriously controversial whether the condition applies. But very often, it is characteristic of such peripheral cases that natural language usage fails to fully determine them.^36^ Even if, say, Causation_M_, Causation_L_ and causation (as used in natural language) all converge on the core cases, there are going to be peripheral cases in which they do not, and furthermore, all the cases we are likely to be at all interested in—as philosophers, or as lawyers and legal theorists—are going to be peripheral cases.

So—as we had already weighty reason to think even before going through the theoretical details—nothing about natural language will make succumbing to the import temptation defensible.

Let me conclude this section with another possible way of mobilising natural language in support of the import temptation. You may think that *the legislature* speaks in natural language, and for this reason, courts, which in some sense owe their allegiance to the legislature, must make sure that, say, Intention_L_ does not depart too widely from (natural-language) intention.^37^ Now, this line of thought raises profound issues—for instance, regarding legal interpretation and what courts owe legislatures—that I cannot discuss here. But it is important to see that we can bypass these complications here. In order to be relevant to our discussion, the natural-language manoeuvre has to bridge the gap between philosophy and law, between (eg) Intention_P_ and Intention_L_. And it is highly doubtful that the legislature is invested in some fancy theory in the philosophy of action, the kind of thing that can constitute exciting breaking news from the philosophy-of-action seminar room. Whether this breaks the connection between Intention_P_ and intention, or between intention and Intention_L_, may depend on those deeper issues that above I postponed for another occasion. But as long as at least one of these connections is broken, the result is the same—the political version of the natural-language argument in favour of the import temptation cannot succeed.

## 4. How Can Philosophy Help?

If everything thus far said is correct, how *can* philosophy be of value to the law and to legal theory? One is tempted to say that philosophy is of value when it is, not because of its implications or policy recommendations. It is of value in itself. And I agree with this. (Have I mentioned that I am a philosophical imperialist?) So the mere fact that philosophy cannot—if indeed it cannot—result in recommendations to a judge or a legislator falls short of showing that it is of no value, and this applies to the philosophy of law as much as to any other kind of philosophy. But the situation is much more worrying: recall that the problem with a philosophy of law that fails to appreciate Catherine’s insight and succumbs to the import temptation is not just that it cannot give sound practical recommendations, but that it cannot promote our understanding of the law. Surely, if philosophy of law cannot do *that*, it is not worth doing.

But once it is understood that this is the problem, the way forward with solving it seems clear. We should philosophise about the law, all the while fully internalising the fact that the law writes on the susceptible skins of living beings. This means we—who write on patient paper—will have to resist the import temptation. But it does not mean that we will not have anything of value to say. In the following subsections, I point to ways in which philosophy of law may be of value. The list does not aspire to exhaustiveness: I think of the following subsections mostly as examples of how a philosophy of law that fully realises Catherine’s insight can nevertheless be of value.

### A. Negative Contribution

In many contexts, many legal ones included, there is no option of not doing philosophy. The only alternatives are doing philosophy well or doing it poorly. And as Hillary Putnam says,^38^ ‘the problem with just giving up on philosophy is that bad philosophy is omnipresent’. So here is one contribution philosophy of law can make, fully realising Catherine’s insight: it can at least expose the mistakes of the philosophers who do not.^39^

This, I think, can be an important contribution. (I hope that this article plays a role in making such a contribution.) But I also want to note how depressing it would have been had this been the only contribution legal philosophers can make—undermining the theoretical and practical harm done by their fellow legal philosophers. So I am happy that this is not where we stop.

Somewhat more positively—but still rather negatively—a legal philosophy that fully internalises Catherine’s insight can help when it is also legal practitioners—not just legal philosophers or theorists—who are tempted by the import temptation. Perhaps, for instance, it is not just philosophers of law and some legal theorists, but also some judges and other legal practitioners, who are tempted by the import temptation when it comes, say, to knowledge and evidence. And perhaps this can explain some of their views and practices when it comes to evidence law (and not just there). We already know that such an explanation can only be a debunking explanation—because when it comes to *justification*, the import temptation should be resisted. But debunking explanations can be helpful in promoting understanding, as they help in undermining support for some views, thereby making others more plausible. Perhaps, for instance, the general antagonism towards statistical evidence—among legal practitioners and theorists alike—can be explained by pointing out that statistical evidence is often not sufficient for knowledge (that is, if we have to, for Knowledge_E_), together with the realisation that when it comes to knowledge, legal practitioners and legal theorists alike do not resist the import temptation. If this is so, this can serve to debunk (at least partly) the thought that statistical evidence should indeed be legally suspicious.^40^ This, of course, would be a genuine contribution. Indeed, it would even be—in this indirect way—practically relevant, *en route* to making, say, suggestions for legal reform.

### B. Lower-Ambition Import

The import temptation is about *entailment*. The temptation is to think that conclusions about (say) criminal attempt *follow* from discussions in the philosophy of action about the nature of trying, and this temptation, I have been arguing, should be resisted. But some less ambitious kinds of import can be defended despite the argumentation of the previous sections.

Thus, thoughts about, say, accounts of meaning in the philosophy of language, while they do not *entail* anything about how meaning and interpretation should be understood in legal contexts, may very well be a good source of insight, or may give legal theorists ideas about a further option that they had not thought about. The reasoning should be understood not as ‘Here’s our best account of interpretation from the philosophy seminar room, so this is what interpretation comes to in legal texts as well’, but rather something like ‘Here’s a promising account of interpretation from the philosophy seminar room, so this is an account that may be worth considering in the legal context as well’. Thus understood, the ideas from the philosophy seminar room—even the breaking news from there!—can be very productive at serving ideas to the legal theorist. But the legal theorist or philosopher still has to do the rest of the work: they have to check the relevant practical considerations—the kind of consideration that governs the law—to see whether the idea that proved productive in the philosophy seminar room is as good when thought of as a part of the law, in other words, whether that idea looks as promising when written on the susceptible skins of living beings as it did when written on patient paper.^41^

### C. Moral and Political Philosophy of the Non-ideal Kind

Some parts of legal philosophy are, as it were, instances of straight up moral and political philosophy, without any import attempts from some other parts of philosophy. Indeed, much traditional legal philosophy is. Questions about the moral bounds of the law (or perhaps the criminal law), about the limits of a duty to obey the law (if such exists), about the best way to design a procedure for our courts or about whether we should try to eliminate the role of luck in our private law system—these questions and many more can be asked without any attempt at illegitimate import. In other words, moral and political philosophy does not lose any of its force or value in those cases in which it is partly about the law.

Still, Catherine’s insight is not without value here either. Catherine, recall, was not even explicitly contrasting *the law* with philosophy. Rather, she was contrasting *her position as a ruler* with that of a philosopher. So it would be surprising if her insight does not apply to moral and, perhaps especially, political philosophy (even when they are not about the law). Let me spend some time, then, on ideal and non-ideal theory.

Political philosophers and theorists often distinguish between ideal and non-ideal normative theory. Ideal theory is done under some, well, idealising assumptions. Perhaps, for instance, you are trying to come up with the most basic principles of social justice. You may want to do this, at least as a first step, for a suitably idealised society—perhaps, for instance, one where everyone is reasonable, or perhaps one where the principles of justice, whatever you end up arguing they are, will be fully or almost fully complied with.^42^ Non-ideal theory avoids such idealisations, and attempts to find the answers to the normative questions it asks given the conditions of reality, including social reality, as we empirically know it. So, for instance, if you are trying to find the basic principles of justice but doing non-ideal theory, the level of compliance you will be assuming is the level of compliance that is likely, based on empirical evidence, to occur. Quite a lot has been written about ideal and non-ideal theory—how best to understand the distinction (beyond the initial characterisation above), which kind of theory political philosophy should be mostly about, and so on. There is no need to engage this literature in detail here. All we need (pretty much) is the following, rather obvious truth^43^: it is perfectly okay to do ideal theory. And it is perfectly okay to do non-ideal theory. But it is not okay to do ideal theory and then pretend that your conclusions are relevant, as they are, to the real world, as if you have been doing non-ideal theory all along. You can do your ideal theory and write it on paper, because paper is patient. Politicians, though, and the law write on the susceptible skins of living beings, and for this reason they have to make sure what they do is governed by the normative considerations that appreciate this. If politicians or legislatures try to directly implement principles that are true only as a part of ideal theory, that may be disastrous. A good political philosopher fully realises this fact, and—to the extent that they are interested in offering practical guidance—makes sure to do this only on the basis of non-ideal theory.^44^

In the ideal theory literature, analogies are often drawn with idealisations in science. For a good case, consider how we sometimes do Newtonian mechanics assuming frictionless surfaces.^45^ If you are assuming frictionless surfaces, you are, of course, doing ideal theory, you are idealising friction away. And it is okay to do so, because you are writing on paper, and paper is patient. Engineers need, of course, to assume more realistic assumptions about friction when they are designing roads (say), because engineers write on the susceptible skins of living beings. Still, it is clear that when you do frictionless mechanics, you often get an ‘a-ha’ feeling of understanding all sorts of real-world phenomena as well. The no-friction idealisation seems to let us see better other, deeper, real-world regularities and explanations, rather than obstructing our view. Perhaps in this way, then, doing frictionless mechanics, on patient paper, can be of some use even to engineers, who ultimately write on the susceptible skins of living beings. But it would make no sense to try and do ‘ideal theory aerodynamics’ by idealising air resistance away. The problem is not just that aeronautical engineers—who write on the susceptible skins of living beings—cannot settle for such ideal theory. The problem is that air resistance is part of what defines the very problems and issues that aerodynamics discusses. For this reason, air-resistanceless aerodynamics is not a cleaned-up version of aerodynamics, it does not let you understand better some real-world phenomena. Rather, if it makes sense at all, it amounts to a radical change of subject. By assuming air resistance away, one does not clean up aerodynamics; rather, one leaves it behind and proceeds to do something else.

So the thing to ask about legal philosophy is whether, by doing ideal theory, we are cleaning up some important question and can still gain insight into the real-world problems we started with, as when we are doing Newtonian mechanics with frictionless surfaces; or are our idealising assumptions more similar to assuming away air resistance and still pretending we do aerodynamics? I do not think this question has a general, uniform answer.^46^ This is a question that has to be asked about every specific case of idealisation in legal philosophy.

Much of the discussion in the literature on ideal and non-ideal theory is on the purported *priority* of ideal theory over non-ideal theory.^47^ What I want to emphasise here, though, is that, at least when it comes to legal philosophy, there is a sense in which *non-*ideal theory is prior. The law, as a matter of its very essence, regulates and so affects the lives of real people, it writes on the susceptible skins of living beings. So this is a part of what defines the problems we are interested in when we are doing philosophy of law. Ideal theory may yet be productive—it may be a useful tool, making us better prepared for doing non-ideal theory. Or it may in some other way shed light on the problems we are interested in. But if any kind of ideal theory is worth doing in the normative parts of the philosophy of law, this is so because it sheds light on the problems as understood in non-ideal theory. Legal philosophy must be done, as it were, with Catherine in mind—if it is of no possible interest to an unfortunate empress who writes on the susceptible skins of living beings, it is probably not good philosophy *of law*.

### D. Bridging Principles

Recall how disambiguation helps with resisting the import temptation: once we suitably distinguish, say, between Causation_M_ and Causation_L_, it becomes clear that no statement about the former entails one about the latter. Well, not *by itself*. But with the right auxiliary premises, it may. If, for instance, we have some independent reason to think that Causation_M_ should matter legally, this may change. Perhaps we have some independent reason to think that it would be wrong to hold someone liable in torts for a harm that they did not *cause*_*M*_. If so, then news about Causation_M_ will have legal consequences. Indeed, depending on the details of this auxiliary premise, it may bridge the gap between Causation_M_ and Causation_L_. I will call such auxiliary premises ‘bridging principles’. The schematic picture, then, is that no statement about Causation_M_ entails all by itself a statement about Causation_L_, but that once the premise about Causation_M_ is conjoined with the right bridging principles, we may get such entailments.

So far, though, a schematic picture is all that we have got. And, indeed, we already have some reason to be sceptical that many interesting realisations of this scheme are forthcoming. This is so because of the breaking-news-from-the-seminar-room scenario I have been ridiculing. If a suitable bridging principle—say, bridging the gap between Causation_M_ and Causation_L_—is independently plausible, then judges *should* be sensitive to the breaking news from the metaphysics seminar room. But it seems highly unlikely that they ever should. So no such bridging principle is plausible.

Perhaps, though, this way of generalizing would be too quick. You may have noticed that the examples I have been using—of news from different seminar rooms—did not include a moral and political philosophy seminar room. This was because—and this is related to the previous subsection—I think that news from those seminar rooms may indeed require the attention of legal practitioners and theorists alike.

Think, for instance, of the (purported) principle according to which it is wrong to convict and punish someone for an action unless they are blameworthy for it. Or perhaps consider the somewhat stronger proportionality principle, according to which it is wrong to punish someone for an action, even if they are blameworthy for it, in a way that is disproportionate to *how* blameworthy they are for it. Or perhaps a contributory negligence rule—in torts—that is put in terms of fault and responsibility. These are principles that aspire to govern criminal law and private law. But the reference to blameworthiness or responsibility in them is most naturally read as reference to moral blameworthiness and responsibility, to Blameworthiness_M_ and Responsibility_M_. So such principles—very traditional and plausible ones in criminal law and tort law theory—are precisely realisations of the scheme of bridging principles I have been talking about. And what their plausibility entails is that judges (and legislatures, jurors, prosecutors …) *should* be attuned to relevant breaking news from the moral philosophy seminar room. If we come to understand moral blameworthiness and responsibility better in some crucial respect, *and if we have independent reason to believe that moral blameworthiness should govern criminal punishability and that moral responsibility should sometimes govern liability in torts*, then we definitely *should* revise our views regarding punishability and liability—and our punishability and liability *practices*, in general or in some specific case—in light of the new understanding of moral blameworthiness and responsibility.

If this is right, then when it comes to blameworthiness, there *is* a plausible bridging principle that makes the relevant part of philosophy relevant to the law. So this is an instance where philosophical content—here, from moral philosophy—can be imported into the law and legal theory without committing the fallacy involved in succumbing to the import temptation. And there are other plausible examples. Perhaps, for instance, we have some independent reason to think that the legal authority of some institution depends on whether that institution represents the governed. If so, then breaking news about the nature of representation and the conditions necessary for one thing to represent another—the kind of stuff that may be discussed in the political philosophy seminar room—may be relevant to constitutional law and constitutional law theory. The claim about authority and representation is the needed bridging principle here, and while I am not sure whether anything like it is true, it is certainly a legitimate contender. So if there is a plausible argument for this bridging principle, conclusions about representation can be imported back into the law.

Even in these cases, though, we should be open-minded about the following possibility: even an initially plausible bridge principle may have to be discarded later on. In particular, if the thought that a new understanding of moral blameworthiness should affect our judgments about justified punishability—in general, or in some specific case—seems too counterintuitive, this in itself may be a good enough reason to revise and in some cases come to reject the bridge principle we thought initially plausible. As is always the case with valid inferences, the plausibility of the premises may confer respectability on the conclusion, but similarly, the implausibility of the conclusion may confer irrespectability on (one of) the premises. Whether we should *modus ponens* our way from the premises to the conclusion or *modus tollens* our way from denying the conclusion to denying one of the premises is something that should be decided on a case-by-case basis, according to judgments of overall plausibility.

These examples of bridging principles—about blameworthiness and representation—were from ethics and political philosophy. But I am not sure this is a necessary feature of all plausible bridging principles. Return to blameworthiness, but now consider a possible relation between blaming and knowledge (that is, between blaming_M_ and Knowledge_E_).^48^ For it may be argued that it is wrong to blame someone unless we *know* that they acted wrongly and indeed culpably. And it may be further argued that it is wrong to punish someone unless it is permissible to blame them.^49^ If so, we have here a complex bridging principle—it ultimately bridges the gap between knowledge and criminal liability, and it does this via thoughts about justified blaming. But an indirect bridge is still a bridge.

I am not sure what I think of this suggestion. The thought that knowledge matters to the law (here, to evidence law, or to the appropriate burden of proof in criminal law) indirectly, by virtue of mattering to justified blaming, seems to me *much* more plausible than the thought that knowledge matters to the law directly, so this is progress. But it is not clear to me how significant this is. For on the current suggestion too, courts ultimately have to be sensitive to the breaking news coming out of the epistemology seminar room. And this remains, in my opinion, as implausible as it always was. Perhaps this is some evidence that, in at least some cases of this kind, we should take—in accordance with the point from two paragraphs ago—the implausibility of this result as a reason to reject the initially plausible-sounding bridging principle.

Be that as it may, the main lesson of earlier sections still stands: no import is licensed without an independently plausible bridging principle. And this is important. Consider heated debates about constitutional interpretation. A view may be argued for by claims about what the meaning of a constitutional clause is or how it ought to be interpreted based on claims about what meaning *is*, perhaps referring to philosophical accounts of meanings or to natural language. Such moves can be quite rhetorically effective. But, we now know, as they stand they must be resisted: if need be, we can distinguish between Meaning_L_ and Meaning_P_ (and natural-language meaning, or Meaning_NL_), and the inference is blocked. If it is to be restored, this must be by answering the question why it is that the law should care about Meaning_P_ or Meaning_NL_. And doing that will amount to defending an independently plausible bridging principle—say, that constitutional interpretation ought to track Meaning_P_. I doubt that such a principle can be defended, but perhaps it can. The important thing here is that if the move above is to be salvaged, something like this bridging principle has to be defended. And this means that the discussion—the real discussion—to be had here is not, certainly not primarily, about the nature of meaning. Rather, it is a normative discussion about what considerations ought to guide constitutional interpretation. *This* is the discussion that all parties should engage in. No one gets to win, normatively speaking, for free, without engaging in normative argumentation, simply on the basis of some claims about the nature of meaning.

Similarly, of course, for all other cases of import. Insisting on the need for substantive, normative, independently plausible bridging principles makes the need for normative argumentation clear and explicit. This alone makes it important—in many contexts—to resist the import temptation and to insist on the necessity of bridging principles.

## 5. Going Wider

So far, this article has been about whether and how philosophy should matter to the law. But we can expand our inquiry. What, more generally, should matter to the law? What should matter to other things, like to morality? To what things should philosophy (metaphysics, epistemology, philosophy of action …) matter? Perhaps fully generally, what should matter to what? Such generalisations are important not only in themselves, but also because they may be seen as the beginning of an objection to what I have been arguing for. Subscripts, after all, come very cheap. We can disambiguate to no end. By the same reasoning I employed above to argue that the import temptation should be resisted, does it follow, then, that nothing should matter to anything else? If so, that would be a very weighty reason to think that something has gone wrong in my argument against the import temptation.

‘What should matter to what’ is a bit *too* general a question to embark on this late on in the article. Instead, I want to briefly comment on two important instances of this general question that go beyond what has already been discussed: whether empirical science should matter to law and whether metaphysics should matter to morality.

First, then, science and the law. Think causation again. Science is to a large extent about studying causal relations—what causes what under what circumstances. And it is natural for the law to use lessons about causation that come with the backing of science (or perhaps of *some* sciences). Is this, though, a practice that is threatened by the reasoning above? Should we distinguish between Causation_S_ and Causation_L_, and insist that while the scientific evidence can establish the former, it is strictly speaking irrelevant to the latter? This, it seems, would be a very troubling result, for without a doubt, the law *should* rely on science^50^ when it comes to such claims about causation. Indeed, the law’s ability to supply whatever goods it is supposed to supply seems to depend on the law’s relying on science (no similar claim is plausible, say, regarding metaphysics).

I do think that such disambiguation is theoretically called for. Usually, though—and in this respect, unlike the case of philosophy discussed in previous sections—it will not be practically needed. The reason for this is that the bridging principles needed to license the import of science into the law are for the most part obvious and uncontroversial. That at least most of the time the law should not pronounce something a cause of something else unless it is, according to our best science, a cause of it seems like something that in most contexts we can take for granted. And when an auxiliary premise is obviously true, we tend to neglect it in presenting arguments, without any danger or harm. And plausibly, this is what explains why it seems like there is no need for the bridging principle, why even the inference without it seems pretty good-looking: because the auxiliary premise needed to make it genuinely good is one we often take for granted. So yes, Causation_S_ and Causation_L_ are not necessarily identical. Inferences from the former to the latter are never complete. But with an entirely obvious auxiliary premise, they often are. So all is still well. This, I think, is true about most cases of importing lessons from science to the law. There is a need for bridging principles, but these bridging principles are rather obvious.^51^

But I also want to remind you that such bridging principles need not apply in full generality. And it is in no way unheard of for a judge, or indeed a legal doctrine, to insist in some legal contexts on separating Causation_M_ and Causation_L_. Perhaps, for instance, they will insist that for some purposes what is needed for legal causation is that one event is *the proximate* cause of another, so that being a cause_S_ does not suffice for being a cause_L_. Or perhaps legal doctrine can insist that in some cases (of joint enterprise, perhaps^52^) cause_S_ is not even necessary for cause_L_. There is nothing wrong, then, with the disambiguation manoeuvre, and it may even make things clearer theoretically. It is just that most of the time, for practical purposes, it is not useful.

Thoughts about how science should matter to the law, then, cannot ground an objection to the argument above about how philosophy should (and when it should not). The structure of that argument applies to the science case as well. In this respect, the argument does generalise. But because the needed bridging principles are for the most part obvious, this generalisation does not have any problematic implications. The argument applies in this wider context, then, but is much less interesting in that context.

How about whether metaphysics should matter to *morality*? Think, for instance, of our practices of holding each other morally responsible, and of metaphysical discussions of freedom of will. It is often assumed that responsibility presupposes freedom. Does this mean, though, that we should all await any breaking news from the metaphysics seminar room, in order to know whether or not to hold someone responsible when they mistreat us? Or should we distinguish between Freedom_M_ and Freedom_E_ (for ‘ethics’; it is a shame that ‘metaphysics’ and ‘morality’ both start with an m), and insist that while Freedom_E_ is a necessary condition for moral responsibility, no conclusion about Freedom_M_ is strictly speaking relevant to moral responsibility? Perhaps we should disambiguate in this way, and defend some bridging principle between Freedom_M_ and Freedom_E_?

I am not sure what exactly to say about this case, except to insist that here too there is room in logical space for such disambiguation, and the moral significance of metaphysics cannot be taken for granted. Here as everywhere else, normative questions are only answered by normative arguments.^53^ If a metaphysical fact is morally relevant, this is in virtue of a moral bridging principle making it relevant. And I am not sure what bridging principles exactly are defensible in the vicinity of moral responsibility and a more metaphysical notion of free will (indeed, the implausibility of waiting, with our responsibility practices, for the breaking news out of the metaphysics seminar room may be an indication that some of the possible bridge principles in this vicinity are false).^54^ Still, it is important to note here too the need for such bridging principles, because often the way to philosophical progress—and certainly to finding new, hitherto unknown areas of logical space—is precisely to question a bridging principle that so far we have been taking for granted.^55^

## 6. Conclusion, and What Have I Been Writing This Article on?

By way of a recap, let me emphasise the take-home lessons that, if successful, this article has established. First, the import temptation and the fallacy it brings along with it is to be avoided. Second, a specific case of import may be justified if supported by the right kind of bridging principle. But it is always a good question to ask what exactly the needed bridging principle is, and why we should believe it. One of the main payoffs of noticing the import temptation for the fallacy that it is, is that it makes it necessary to state explicitly the needed bridging principle and to defend it substantively, normatively. Thus, one of my hopes for this article is that it will make it harder for some to hide their implicit (and possibly controversial and problematic) bridging principles behind a seemingly obvious, indeed linguistically required, import. Third, none of this is a problem for the philosophy of law in general. There are ways of doing legal philosophy that in no way involve illegitimate import. The trick, then, is not to avoid the philosophy of law, but to do it well.

But now we can also ask: if law, as I have been insisting, writes on the susceptible skins of living beings and philosophy typically writes on patient paper, what does the philosophy of law write on? What have I been writing this text on?

Philosophy of law is about the law, it is about something that writes on the susceptible skins of living beings. But it is still an instance of philosophy. We write on patient paper, we do not directly affect the lives of real people. Practical considerations do not directly apply to what we do—in philosophy of law, as elsewhere in philosophy—which is why we can let theoretical or epistemic considerations rule. And this applies to this text as well.

This too is important: if you attempt to make legal decisions or to do legal theory without realising that the law writes on the susceptible skins of living beings, you will be making bad law or doing poor legal theory.^56^ Similarly, if you are doing philosophy of law without realising that you are writing on paper which is patient, you may be doing poor philosophy. Some philosophers write as if what they write will immediately change the real world, and so give more weight to practical considerations and less to theoretical ones in their writing.^57^ This is the same mistake as that underlying the import temptation, except, as it were, in the opposite direction. It is another failure to understand that different projects are governed by different normative standards.

Of course, distinctions in the real world are rarely as clean as they are in philosophical texts. There may be borderline cases of texts in the philosophy of law that also have—perhaps in virtue of the immense influence of their writer on decision makers or the general public—significant effects on the lives of real people. Perhaps this would be a case in which someone is trying to write simultaneously—and the very same text—both on patient paper and on the susceptible skins of living beings. (Or perhaps this is where Catherine’s metaphor ceases to be helpful.) In such cases, some balance between the different governing standards will have to be struck and, quite plausibly, practical considerations will be dominant.

Fortunate academics that we are, however, not many of us have the kind of influence that would be needed to generate such dilemmas. We can be quite content writing on paper. But we should fully internalise the fact that it’s not paper that the law writes on.

## Footnotes

1 There are different versions of this quote on the web, as you would expect. The reference is often to Catherine’s correspondence with Diderot. For a closely related quote, with a reference to Louis-Philippe de Ségur’s Mémoires, see R Zaretsky, *Catherine & Diderot: The Empress, the Philosopher, and the Fate of the Enlightenment* (Harvard UP 2019) 224. There is a similar quote (in French) in Maurice Tourneux, *Diderot et Catherine* II (Calmann Lévy 1899) 81.

2 As do scientists, of course. But they typically ask what causes what, not questions about the nature of causation. I return briefly to discussing science in section 5 below.

3 Alex Kaiserman, ‘Partial Liability’ (2017) 23(1) Legal Theory 1; this follows from the more purely philosophical Alex Kaiserman, ‘Causal Contribution’ (2016) 116(3) Proceedings of the Aristotelian Society 387.

4 Following Susan Haack, *Evidence Matters: Science, Proof, and Truth in the Law* (CUP 2014).

5 For a discussion that, on the one hand, seems to assume that what we learn about intentions in the philosophy of mind will be relevant for ethics and the law, but that, on the other hand, is often careful about asking whether this category should matter, see Kimberly Kessler Ferzan, ‘Beyond Intention’ (2008) 29 Cardozo L Rev 1147.

6 I am making this assumption in order to abstract away from questions of purer fact finding.

7 This is a rough initial statement of the causal theory of knowledge, which used to be influential. For one *locus classicus*, see Alvin Goldman, ‘A Causal Theory of Knowing’ (1967) 64(12) The Journal of Philosophy 355. It no longer is. For a survey of the view and its fate, see Marshall Swain, ‘Causal Theory of Knowledge’ in E Craig (ed), *Routledge Encyclopedia of Philosophy*, vol 5 (Routledge 1998) 263–6.

8 This is a rough statement of the main thesis of pragmatic encroachment. For an overview, see Brain Kim, ‘Pragmatic Encroachment in Epistemology’ [2017] Philosophy Compass 12.

9 This ‘all by itself’ qualification is important—see the discussion of bridging principles below.

10 You may think that this is so because ultimately, even when the law uses the term ‘knowledge’, that is not what it means. Indeed, often the ‘knew or should have known’ condition can be legally satisfied, say, by a merely true belief. At other times, however, the law does seem to be about knowledge. See eg the discussion of Gettier cases in David Enoch, Talia Fisher and Levi Spectre, ‘Does Legal Epistemology Rest on a Mistake?’ (2021) 31 Philosophical Issues 85.

11 Note that the intuition remains strong—for me, just as strong—even if the news from the epistemology seminar room is (miraculously) that a full consensus among epistemologists has emerged on the relevant point. So, to repeat a point I made in the introduction, the problem is not merely the absence of philosophical consensus.

12 Famously, in *McGhee v National Coal Board* 1973 SC (HL) 37, 53, Lord Reid observed that ‘it has often been said that the legal concept of causation is not based on logic or philosophy. It is based on the practical way in which the ordinary man’s mind works in the everyday affairs of life.’

13 Below, I qualify this statement, but not by much.

14 In Enoch, Fisher and Spectre (n 10), we for the most part put this point by saying that the law should not care about knowledge, that knowledge (and epistemology) does not matter to the law. We did this because we took it for granted (again, for the most part) that knowledge is the kind of thing that epistemologists study. But the disambiguation in the text allows me to make this point more precisely now.

15 For a discussion of causation that explicitly distinguishes between the interests of philosophers and lawyers—though along different lines than the ones I emphasise in the text—see Jane Stapleton, ‘Choosing What We Mean by Causation in the Law’ (2008) 73 Mo L Rev 433.

16 For similar claims regarding the philosophy of action, in the context of criticising Yaffe’s discussion of attempts, see David Enoch, ‘Comment on Yaffe’s *Attempts*’ (2012) 6(1) Jerusalem Rev Leg Stud 20. In his reply, Yaffe clearly goes for the bridging-principle strategy, the one I discuss in section 4D below. Gideon Yaffe, ‘The Legal Importance of Trying: Reply to Enoch, Dahan-Katz, and Berman’ (2012) 6 Jerusalem Rev Leg Stud 51, 57—though not, I think, in the original book, Gideon Yaffe, *Attempts: In the Philosophy of Action and in the Criminal Law* (OUP 2010). See also Yaffe’s praise for Moore’s work, where Yaffe seems to explicitly attribute to Moore (what I would call) succumbing to the import temptation. Gideon Yaffe, ‘Moore in Jeopardy, Again’ in Kimberly Kessler Ferzan and Stephen J Morse (eds), *Legal, Moral, and Metaphysical Truths: The Philosophy of Michael S Moore* (OUP 2016) 111.

17 In Enoch, Fisher and Spectre (n 10), we put the point in terms of such questions as how many more innocents are you willing to have your system put in jails just in order to satisfy some philosophical niceties about how you use words?

18 In Enoch, Fisher and Spectre (n 10), we briefly consider (without endorsing) a two-tier system where, even though considerations from epistemology should have no weight as a part of system design, they may have some weight for the conscientious judge making a specific decision. Here, I go more clearly against this possibility—and if even the judge should not give such considerations any weight, this applies even more clearly to system design.

19 For very similar points in discussing the relation between Jewish law and metaphysics, see Aaron Segal, ‘Metaphysics out of the Sources of the Halakha, or Halakhic Metaphysics?’ in Samuel Lebens, Dani Rabinowitz and Aaron Segal (eds), *Jewish Philosophy in an Analytic Age* (OUP 2019) 34–59. His context is different—he is more interested in whether metaphysical views can be attributed to the Jewish law; his arguments are different, being mostly based on the different methods of deciding disagreements about legal and metapyhysical issues; and I am, of course, not committed to the Halakhic-specific parts of his discussion. Still, on the central point in the text here, Segal is a kindred spirit.

20 See David Enoch, Levi Spectre and Talia Fisher, ‘Statistical Evidence, Sensitivity, and the Legal Value of Knowledge’ (2012) 40 Philosophy and Public Affairs 197; David Enoch and Talia Fisher, ‘Sense and Sensitivity: Epistemic and Instrumental Approaches to Statistical Evidence’ (2015) 67 Stan L Rev 557. We emphasised the lesson drawn here in Enoch, Fisher and Spectre (n 10). For a helpful recent survey, see Lewis Ross, ‘Recent Work on the Proof Paradox’ [2020] Philosophy Compass 15.

21 Thomson was an influential early contribution in this spirit. See Judith J Thomson, ‘Liability and Individualized Evidence’ (1986) 49(3) LCP 199.

22 See eg Clayton Littlejohn, ‘Truth, Knowledge, and the Standard of Proof in Criminal Law’ (2017) 197 Synthese 5253; Sarah Moss, ‘Knowledge and Legal Proof’ (2023) 7 Oxford Studies in Epistemology 176.

23 See Michael Blome-Tillmann, ‘“More Likely Than Not”—Knowledge First and the Role of Statistical Evidence in Courts of Law’ in A Carter, E Gordon and B Jarvis (eds), *Knowledge First—Approaches in Epistemology and Mind* (OUP 2017) 278–92. To an extent, see also Liat Levanon, ‘Statistical Evidence, Assertions and Responsibility’ (2019) 82 MLR 269.

24 Unless, that is, there is some other story—of the kind that I call below ‘bridging principles’—that can render it relevant.

25 There are similarities between the points in the text here and Julie Dickson’s ‘indirectly evaluative legal philosophy’. Dickson goes even further. She thinks that the *methodology* we use in doing legal theory and philosophy should be sensitive to the practical significance of law. On this, I differ. I do not see that the methodology of legal philosophy is or should be different from that of any other kind of philosophy. It is just that we should always be sensitive to the nature of what we philosophise about. See Julie Dickson, *Elucidating Law* (OUP 2022).

26 The context of close things said by Catherine to Diderot makes this reading at least a plausible interpretation. Again see Zaretsky (n 1) 224. I thank Luba Gornstein, who corresponded on my behalf with the National Library of Russia in St Petersburg. They found similar texts to the ones quoted by Zaretsky. And I thank Rivka Klein for finding the text in French that Zaretsky is referring to. (Both Luba and Rivka are from the Hebrew University libraries.)

27 Robert Cover seems to express a similar idea: ‘Legal interpretation takes place in a field of pain and death.’ See Robert M Cover, ‘Violence and the Word’ (1986) 95 Yale LJ 1601, 1601. A similar idea is also a central theme in Dickson (n 25) (though she tries to make use of it in the context of general jurisprudence, and I do not want to commit myself to *that*).

28 Note that at no point do I assume a consequentialist framework. The set of practical considerations that should govern the law here may include deontological ones.

29 The claim that the law writes on the susceptible skins of living beings—that it affects the lives of real people—is perhaps as close as we can get to a conceptually necessary truth about the nature of law. It is in some sense weak—presumably, this is not unique to the law, so this condition is not sufficient. In another sense, though, it is strong: it is not just a conceptually necessary condition for something—a system of rules, perhaps—to count as law, it is also an important one, one that you cannot miss without getting the nature of law seriously wrong.

30 Do Hart and Honoré, for instance, commit a fallacy by taking the everyday notion of causation, presumably the one studied by metaphysicians, to be a guide to legal causation? Or are they endorsing a principle that bridges the gap between the two, and indeed best seen as arguing for it? HLA Hart and AM Honoré, *Causation in the Law* (Clarendon Press 1959). See also n 16 above, about Yaffe’s endorsing the bridging-principle strategy in response to my earlier criticism of his book.

31 I thank Julie Dickson for a discussion of the point in the paragraph in the text.

32 [1978] FH 7/78 *Zaktzer v State of Israel* 32(2) PD 828.

33 Does the law’s role in guiding behaviour not entail that it must use terms in their natural-language senses? Well, fulfilling a guiding role requires comprehensibility. But comprehensibility does not require using terms—certainly not all terms—in their natural-language senses. One can use a different idiolect, as long as those one is supposed to guide know that this is what one is doing. And often everyone with a legal education knows that. As for guiding ‘the common folk’, often the law achieves this indirectly, in a way that is mediated by those who speak the idiolect.

34 The point in the text may be read as a cheap shot at the currently influential ‘conceptual engineering’ programme. Now, I do not think that the point in the text suffices to refute that programme—to claim to do that it would have to presuppose an oversimplified version of conceptual engineering. But in the name of intellectual honesty, I want to note that I am not a fan of that programme, and that my reasons for not being a fan *are* related to the point in the text. But this is not the place to develop that discussion.

35 For the claim that nothing perfectly deserves the name ‘value’, and how he nonetheless thinks that his specific theory is the best candidate, see eg David Lewis, ‘Dispositional Theories of Value’ (1989) 63 Proceedings of the Aristotelian Society 89, 136) (I differ regarding values, but not regarding the methodology.) I want to address another worry here (for which I thank Ram Rivlin): suppose you are wondering whether there is milk in your fridge. A certain metaphysical nominalism—according to which, roughly, the only things that exist are basic particles—is not relevant. If someone tells you that there is no milk in the fridge, simply because they think milk does not exist, they have not responded to your question in a relevant way. The worry is that the main point of this article is just a particular instance of the wider phenomenon exemplified by the milk example. And it is relevant here because it is about the question whether the philosophical sense of ‘there is’ and the everyday sense (as in ‘is there milk in the fridge?’) are the same sense. Yes, at a sufficient high level of abstractions, the law case and the milk case are similar. Both exemplify how what we are concerned with in different contexts may shape the concepts we use in those contexts. But this level of abstractness may be too high to be helpful, and in some contexts—including ours—it is important to point out precisely how the relevant concept-shaping concern varies across specific contexts.

36 Some people think that language use can give rise to correctness conditions that are much, much more precise and detailed than you would expect. See here Timothy Williamson, *Vagueness* (Routledge 1994). I am not sure I find such arguments convincing, but anyway, we can safely ignore them here. Even if the only problem with peripheral cases—as Williamson seems to think about borderline cases of vague terms—is that it is *unknown* and perhaps also *unknowable* whether or not the relevant concept applies, this suffices for my purposes in the text. It would just require a change in some wording.

37 For a version of this point, see Cosmin Vraciu, ‘Authority, Democracy, and Legislative Intent’ (2024) 43 Law and Philosophy 89. Note, though, that his discussion is in the context of the role of legislative intent. Still, he claims that the same practical reasons that justify the democratic authority of legislation in general also require that the judge apply the public, natural-language meaning of legislation. Note also, however, that Vraciu thinks this lesson only applies in cases in which the natural-language meaning is clear.

38 Phillip McReynolds, Interview with Hilary Putnam, Harvard University, ‘On the Fact Value Dichotomy and Bad Philosophy’ (2013) <www.youtube.com/watch?app=desktop&v=oLJfEVu3kbY> at 1:50 minutes.

39 And, of course, if someone is attempting an import from *bad* philosophy, it may be useful to point that out as well.

40 This is a part of what we are doing in Enoch, Spectre and Fisher (n 20). But then we proceed to put forward an alternative, practical justification of the differential legal treatment of statistical and other evidence.

41 Philosophers of science sometimes distinguish between the context of justification and the context of discovery. The point in the text here is that even if the arguments against the import temptation show that philosophy (of the kind thus far discussed) cannot play a role in the context of justification of legal arrangements, it may still play an important role in the context of discovery.

42 This is inspired by John Rawls, *A Theory of Justice* (Harvard UP 1971), but I do not want to commit myself in any way regarding Rawls’s exegesis.

43 Well, it *should* be obvious. I say more about it in David Enoch, ‘Against Utopianism: Noncompliance and Multiple Agents’ (2018) 18 Philosophers’ Imprint 16, Appendix.

44 The distinction between ideal and non-ideal theory is usually discussed in the context of political philosophy. However, an analogous distinction applies in (non-political) ethics, and everything I say about the distinction in the text applies to such a wider use as well.

45 A common analogy in the ideal theory literature. I use it Enoch, ‘Against Utopianism’ (n 43). The next couple of paragraphs are based on what I say there. See also the references therein.

46 I argue for an analogous claim regarding political philosophy in ibid.

47 I distinguish between different kinds of priorities that could be relevant here in ibid.

48 See eg Christoph Kelp, ‘The Knowledge Norm of Blaming’ (2020) 80 Analysis 256.

49 See eg Lewis Ross, ‘Criminal Proof: Fixed or Flexible?’ (2023) 73 The Philosophical Quarterly 1077, drawing on Lara Buchak, ‘Belief, Credence, and Norms’ (2014) 169 Philosophical Studies 285. Note that here the suggested relation is not between punishability and blameworthiness, but between punishability and justified blaming. In some contexts, this is a very important difference. For the purposes of my example here, however, we need not elaborate on it. It is just that while there is some plausibility to the thought that knowledge is a necessary condition for justified blaming, there is no plausibility to the thought that knowledge (by those who may blame you) is a necessary condition for your being blameworthy.

50 Or perhaps on *settled* science, or on scientific theories that are not too controversial in the right circles. Everything in the text can be suitably modified to fit such qualification—perhaps, for instance, the judge should not be impressed with the breaking news out of science about a new, exciting theory the cool kids are interested in, but only about the less exciting news that some piece of science is now considered settled.

51 The point can also be put in terms of deferential intentions. I suspect that in most legal cases, when legal practitioners use terms of the kind that is studied by the sciences, they are—or at least ought to be—committed to the relevant bridging principles.

52 I am not saying that this is the *only* way of understanding the relevant legal doctrines.

53 In particular, constitutive answers—about the nature of action and agency, say—do not answer normative questions. This is a part of my point in David Enoch, ‘Agency, Shmagency: Why Normativity Won’t Come from What is Constitutive of Action’ (2006) 115 Philosophical Review 169.

54 Interestingly, in this case there are sometimes constraints in the opposite direction: it may be claimed that our only grip on our metaphysical conception of freedom proceeds via our moral thoughts about responsibility and free will. If this is so, this may count for a stronger conceptual connection between Freedom_M_ and Freedom_E_, but in a different way than is assumed by attempts to import metaphysical facts into morality.

55 Think here, for instance, of Parfit’s metaphysical and ethical discussions of personal identity, and his rightly influential claim that perhaps survival, in the sense necessary for the metaphysics of personal identity, is not of value, not what we should care about. See Derek Parfit, *Reasons and Persons* (OUP 1984).

56 Perhaps the following analogy will help. Science is empirical. Philosophy of science, arguably, is not. But, of course, a philosophy of science that fails to account for science’s empiricality is a very poor philosophy of science indeed.

57 This, I think, is ‘in the air’, as more philosophers, across philosophical sub-disciplines, write in more politically motivated ways. Myself, I am all for political motivations for philosophical inquiries (at least, that is, when these are the right political motivations). But I am deeply concerned about philosophers conducting the philosophical inquiry itself in a way that is governed more by political motivation than by theoretical considerations. This practice seems to me intellectually dishonest, and politically counterproductive to boot. But I digress.

