# Correction to: Law, Philosophy and the Susceptible Skins of Living Beings

**DOI:** 10.1093/ojls/gqaf032

**Published:** 2025-08-22

This is a correction to: David Enoch, Law, Philosophy and the Susceptible Skins of Living Beings, *Oxford Journal of Legal Studies*, 2025; https://doi.org/10.1093/ojls/gqaf022

In the originally published version of the manuscript there was a typographical error in the title. This has been emended in the article to read: “Law, Philosophy and the Susceptible Skins of Living Beings” instead of: “Law, Philosophy and the Susceptible Skins of Living Being”.

